# Brain and Muscle: How Central Nervous System Disorders Can Modify the Skeletal Muscle

**DOI:** 10.3390/diagnostics10121047

**Published:** 2020-12-04

**Authors:** Stefania Dalise, Valentina Azzollini, Carmelo Chisari

**Affiliations:** Neurorehabilitation Unit, Department of Translational Research in Medicine and Surgery, University of Pisa, 56126 Pisa, Italy; vale.azzollini89@libero.it (V.A.); carmelo.chisari@unipi.it (C.C.)

**Keywords:** rehabilitation, skeletal muscle, stroke, multiple sclerosis, Parkinson’s disease

## Abstract

It is widely known that nervous and muscular systems work together and that they are strictly dependent in their structure and functions. Consequently, muscles undergo macro and microscopic changes with subsequent alterations after a central nervous system (CNS) disease. Despite this, only a few researchers have addressed the problem of skeletal muscle abnormalities following CNS diseases. The purpose of this review is to summarize the current knowledge on the potential mechanisms responsible for changes in skeletal muscle of patients suffering from some of the most common CSN disorders (Stroke, Multiple Sclerosis, Parkinson’s disease). With this purpose, we analyzed the studies published in the last decade. The published studies show an extreme heterogeneity of the assessment modality and examined population. Furthermore, it is evident that thanks to different evaluation methodologies, it is now possible to implement knowledge on muscle morphology, for a long time limited by the requirement of muscle biopsies. This could be the first step to amplify studies aimed to analyze muscle characteristics in CNS disease and developing rehabilitation protocols to prevent and treat the muscle, often neglected in CNS disease.

## 1. Introduction

Skeletal muscle can be considered a dynamic tissue, not only for its contractile property and its capacity to generate a macroscopic movement, but because it is able to change its characteristics (size, metabolism or fibers composition) in response to various stimuli. Traditionally, the motor disability in patients with neurological diseases is thought to be solely due to brain injury itself. The muscular structural, metabolic and functional aspects accompanying central nervous system (CNS) disease are addressed in a rather episodic way. Although it is widely accepted that impaired CNS function could differently affect local and systemic tissues function and metabolism, it is unclear whether functional impairments of skeletal muscles (weakness, fatigue and changes in muscle tone) are rather consequences of denervation and disuse or of any other intrinsic mechanisms [1,2]. Research focused on the detailed analysis of the intramuscular component and muscle modifications in chronic neurological diseases and their correlation with clinical severity is limited and, often, patient inactivity remains an important confounder when studying the effects of CNS disease on muscle quality. The knowledge of how an altered interaction between the CNS and muscle tissue can change the properties of the latter is fundamental to address the therapeutic rehabilitation approach. In fact, it is known that through different rehabilitation approaches, personalized on patient impairment, it is possible to modify not only the muscular performance but also its structure down to the molecular level [3].

The concept of muscle plasticity, born in 1979, has developed over time, and the idea of the connection between neurons and properties of innervated muscles (type of fiber and metabolism) has been consolidated in the scientific community [4]. Despite this, the muscle is rarely a target in the rehabilitation approach of CNS disease, and recovery is focused only on central motor control rather than on peripheral muscle. A better knowledge of the underlying mechanisms of structural and functional modifications of the muscle, as well as the potential for reversal with a rehabilitation treatment would allow the creation of specific rehabilitation approaches, avoiding neglecting this important executive organ. To date, different assessment modalities are available to study muscular tissue: non-invasive methods have been added to the common muscle biopsy, which remains the most accurate direct valuation method. Indeed, ultrasonography, dual-energy X-ray absorptiometry (DXA), magnetic resonance (RM) or computerized tomography (CT) can provide information about macroscopic changes and composition of muscle. Furthermore, proton magnetic resonance spectroscopy and high-density electromyography can determine muscle fiber-type, and near-infrared spectroscopy (NIRS) and positron emission tomography (PET) are useful to assess muscle metabolism, hence the need for this review, which aims to narratively summarize the results of the last ten years of research in the field of skeletal muscle modifications determined by three of the most common neurological diseases causing disabilities: Stroke, Multiple Sclerosis (MS) and Parkinson’s Disease (PD). These, among all neurological pathologies, represent the most common cause of disability and consequent request for rehabilitation intervention. Rehabilitation interventions proposed in the treatment of these pathologies often include training and physical therapies known to have an impact on skeletal muscle. Understanding the mechanisms underpinning the impairment is essential for the development of specific and personalized rehabilitative interventions and the muscle could become a promising target of treatment.

## 2. Materials and Methods

The studies analyzed for this review are derived from research focused on a limited time interval, from 2010 to 2020, in order to summarize the most recent discoveries in this area. Previous studies, found through manual searches or prior revisions [5], are not considered in the review tables but, as they represent milestones in the study of muscle modifications in neurological diseases, they are cited in the text and used to support or discuss the recent findings. The search engine used was PubMed, separately for each of the CNS acquired diseases mentioned above: stroke, MS and PD.

The keywords used for the search are as follows:

1. “Stroke” [MeSH terms] AND “skeletal muscle abnormalities” [MeSH terms] OR “skeletal muscle metabolism” [MeSH terms] OR “skeletal muscle fibers” [MeSH terms];
2. “Multiple Sclerosis” [MeSH terms] AND “Skeletal muscle abnormalities” [MeSH terms] OR “Skeletal muscle metabolism” [MeSH terms] OR “Skeletal muscle fibers” [MeSH terms];
3. “Parkinson’s disease” [MeSH terms] AND “skeletal muscle abnormalities” [MeSH terms] OR “skeletal muscle metabolism” [MeSH terms] OR “skeletal muscle fibers” [MeSH terms].

Research was further limited to the English language and human subjects. All types of articles (Case Reports, Clinical Trial, Comparative Study, Multicentric Study, Observational Study, Randomized Controlled Trial) were considered, except for systematic and narrative reviews.

Additional exclusion criteria were full text not available, grey literature and studies that consider performance measures, in which CNS involvement is required, as the outcome of muscular modifications.

## 3. Stroke-Induced Skeletal Muscle Modifications

The residual neurological deficits of stroke survivors often impair function and mobility, predisposing them to inactivity that might contribute to deconditioning, fatigue and functional loss. So, muscle tissue modifications after stroke might be caused by the sedentary and inactive lifestyle with reduced loading of the paretic limbs, but the altered neural activation might cause muscular alterations, too [5]. Little is known about changes in structure and metabolism of stroke-affected muscles, as well as about the relationship between these muscular changes and mechanical properties. Several studies have evaluated the neurological alterations and the consequent neural plasticity [6], but only a few have analyzed changes in motor unit (MU) and in muscle properties. The most described muscle change is muscle atrophy, which can be a consequence of inactivity or atrophy following poststroke denervation. Indeed, stroke survivors show an elevated prevalence of sarcopenia when considering matched age, sex and race of healthy individuals [7]. It was demonstrated that myostatin mRNA expression levels were 40% higher in the paretic than non-paretic muscles and it might be involved in stroke-related muscle atrophy [8]. However, muscle atrophy in the paretic limbs detected after stroke does not explain the marked reduction in strength observed in stroke population: other changes in muscle morphology, such as changes in muscular metabolism, fiber structure or alteration in motor control, might contribute to the after-stroke muscle weakness [9].

As a result of our search, only two studies described the fiber type in stroke patients through muscle biopsies. The muscle fiber morphology of the paretic muscle of chronic stroke patients shows a smaller overall fiber cross-sectional area (CSA), a shift towards a low oxidative type IIX fiber content and a reduced type I and type IIA fiber content [10]. Muscle resistance in affected limbs is likely decreased, because type IIX fibers are more fatiguing than type I and type IIA. These changes lead to impaired muscle performance. So, high-intensity training aimed to increase type IIA fiber percentages might contribute to muscle power and endurance, crucial for functional capacity [10,11]. Strictly related to the morphology of the fibers are other studies that analyze the metabolic aspects of the muscle through the analysis of metabolic products or by radioimaging. Measuring the respiratory exchange ratio and rates of fat and carbohydrate oxidation, it was shown that stroke patients rely heavily on carbohydrate oxidation during prolonged walking, while healthy individuals mostly oxidize fatty acids. Carbohydrate utilization, up to 70%, likely indicates a preferred anaerobic metabolism in stroke patients and potentially limits the ability to walk for a long time [12]. The utilization of fats in muscular aerobic metabolism is known to be influenced by muscle mass, muscle fiber type, capillary density and muscle oxidative capacity [13]. Reduced oxygen extraction capacity in stroke survivors could, in part, be attributed to structural changes in skeletal muscle that can begin as early as 4 h after cerebral infarction. This can develop muscle weakness and atrophy within days, also in the unaffected limb [14]. So, stroke patients show an impaired substrate metabolism compared to healthy controls (HC) with an increased lactate and glycerol production on tissue level, delayed and impaired glucose utilization and a mild increase in energy expenditure. The increased glycolytic and reduced lipolytic activity in poststroke skeletal muscles suggests a reduced oxidative capacity and might indicate a bilateral fiber type shift: Klaer et al. did not find differences in skeletal muscle tissue metabolism between paretic and non-paretic leg in stroke patients [15]. Conversely, a PET study found a reduced muscular activity with a consequent lower muscular metabolism in the paretic muscles of inferior limb, but the contralateral side showed an increased metabolism [16]. The different results could derive from the different assessment method or could be due to the different time elapsed since stroke or different patient functional status (not specified by Klaer et al.).

Other information on poststroke fiber typology may come from studies on MU changes; several studies have been performed to understand how the MU is modified in stroke patients. In most cases, stroke causes paresis and impaired motor control that can lead to spasticity; one potential mechanism underlying such impairment in muscle voluntary activation is an early or late modification of the entire MU. In fact, systematic disturbances of MU pool activation are present in paretic muscles of stroke survivors, and it might potentially contribute to motor impairment. The paretic muscles of stroke patients show a reduction in the number of MU compared to the contralateral side, with a larger amplitude of the outlier surface of MU action potential (MUAP) [17]. High-density surface electromyography (sEMG) on biceps brachii highlighted larger MUAPs in chronic stroke patients, indicating enlarged MU, possibly as a result of reinnervation, since the number of fibers that belong to each MU increases by collateral sprouting. Furthermore, there is a positive correlation between the root mean square (RMS) of MUAP and Fugl–Meyer score, which indicates a relation between MU properties and clinically assessed motor recovery [18]. In these terms, the altered MU activation might be a target of a specific rehabilitative approach: it is shown that a rehabilitative treatment provided by robotic assisted locomotion system (Lokomat) induces a significant increase in firing rate, not accompanied by an increase in strength; this could suggest an effect of training on motoneuronal firing rate that thus contributes to muscle motor control [19].

The normal orderly recruitment, based on MU size, is also disturbed in the affected muscles: the recruitment of larger MUs at higher muscle contraction levels was less evident in the paretic muscles, compared with the contralateral muscles. In addition, the threshold force range for MU recruitment was compressed to a lower level on the affected side, indicating a different type of MU fiber [20]. By measuring changes in the muscle fiber conduction velocity (MFCV), a change in the type of muscle fiber could be suggested. Indeed, the MFCV was found to be inferior in the paretic extensor digitorum communis muscle compared to the non-paretic one in stroke patients. Conversely to the prior findings, the measured displacement might indicate an increase in the proportion of active type I fibers compared to type II fibers, which could result from MU dysfunction, preferential atrophy of type II fibers, as well as a denervation–reinnervation of fibers that occurs in time [21].

The sEMG studies appear to contradict the results of the biopsy and metabolic studies mentioned above. Indeed, the increased activation of low-threshold MUs indicates a relative increase in type 1 MU, while the biopsy results suggest a prevalence of type 2 fiber in paretic muscles. In our hypothesis, these findings could be considered in light of the different samples analyzed. The studies focused their attention on different muscles (large muscles of limbs versus muscles of the hand), on patients with different disease duration, without mentioning the possible effect of drug therapy (i.e., antispasmodic treatment) known to cause rheological changes on the muscles. The studies remain conflicting, probably due to the lack of a stratification of patients, based on a correct functional evaluation. The highlighted alterations could be helpful to design a specific rehabilitation approach, aimed to treat the most affected fibers (i.e., aerobic training), but there are no clinical studies that evaluate the effectiveness of specific treatments targeting muscular outcomes.

Studies of the past decade on skeletal muscle changes after stroke are summarized in Table 1.

## 4. Multiple Sclerosis-Induced Skeletal Muscle Modifications

MS patients complain of muscle weakness and fatigue due to impairments of both central motor skills and intramuscular function. It is still unknown whether the muscle changes that occur in MS are the direct result of centrally mediated changes in MU excitability or indirectly from the reduced physical activity and mobility that typically accompany the disease. Over the years, several studies have shown that the biochemistry and composition of skeletal muscle cells in MS are significantly altered. In fact, smaller skeletal muscle fiber, lower succinate dehydrogenase activity, delayed phosphocreatine resynthesis after isometric exercise, blunted intramuscular metabolic responses during isometric exercise and complex-1 deficiency in skeletal muscle mitochondria were showed [22,23,24,25]. Despite all the studies, in recent years, the muscular aspect in MS has taken a back seat and has been overlooked in the specific therapeutic treatment of the disease. Furthermore, the studies of recent decades remain conflicting and confusing.

Scott et al. [26] studied the signal pattern with sEMG of MS patients and HC. They found an altered neuromuscular activity during voluntary contraction of MS muscles, with an increased MFCV and a reduced sEMG amplitude, measured as RMS. They speculated that reduced activity levels along with reduced strength and slower rate of strength development may be due to the denervation atrophy that occurs in MS. This could, then, have led to a higher percentage of type II fibers, which would justify the faster average MFCV observed [26]. This kind of muscular pattern was observed also by Wens el al. on vastus lateralis biopsy of MS patients [27]. Their samples showed a significantly smaller mean muscle fiber CSA of type I, II and IIA fibers in MS, independently from type and disease severity. The study also showed a tendency towards a higher proportion of type IIA fibers, at the expense of type I fibers, as previously reported [22,27].

The results, however, contrast with one study analyzing muscular metabolism in MS. In fact, Malagoni et al. using NIRS found higher resting muscle oxygen consumption (rmVO2) values in MS patients than HC and greater rmVO2 in lower performing patients compared to better performing patients [28]. Assuming that in MS patients there is a shift towards white fibers with fewer type I fibers [22], Malagoni et al. suggest that type I residual fibers develop increased metabolic activity and increased capillarization as a compensatory mechanism to support resistance to walking and fatigue [28]. Conversely, in a more recent study, with the same assessment method (NIRS), Harp et al. highlighted that MS patients had 40% lower mVO2max compared to the HC after exercise, supporting the hypothesis of a major impairment in oxidative fiber [29].

At the molecular signaling pathways level, Hansen et al. found higher levels of phospho- adenosine monophosphate-activated protein kinase A (phospho-AMPKa) and phospho-mammalian target of rapamycin (phospho-mTOR) in MS than in HC [30]. Phospho-AMPKa is considered to govern the mitochondrial biogenesis, while phospho-mTOR the myofibrillar biogenesis. Authors speculated that increased phospho-AMPKa levels in resting muscles might be due to systemic inflammation and vitamin D deficiency, associated with MS. Surprisingly, although MS was associated with an increased level of phospho-AMPK and phospho-mTOR in resting muscles, exercise tolerance and CSA of type IIA muscle fibers were paradoxically decreased. In fact, the increased level of phospho-AMPKa and phospho-mTOR seems to be correlated with lower exercise tolerance (VO2peak) and a greater level of disability (based on Expanded Disability Status Scale—EDSS). Hansen et al. speculate that an inappropriate downstream signaling after muscle phospho-AMPKa and phospho-mTOR occurs in MS, so mitochondrial and myofibrillar biogenesis are not stimulated enough and skeletal muscle AMPKa and mTOR compensate by increased phosphorylation [30]. It should also be considered that the activation of the mTOR pathway could be a sign of initial denervation in MS, since during denervation, the mTOR pathway was unexpectedly activated, and the expression of genes related to myogenesis were markedly increased [31].

The heterogeneity of the disease and its different forms does not allow to extrapolate univocal data. However, in the majority of the studies analyzed in this review, a type I fiber impairment is described through metabolic and biopsy studies. The only study in which this impairment is not so well described is Malagoni et al. [28], where authors suggest a highly compensative mechanism in increased oxidative metabolism in residual type I fibers. This remains an important missing knowledge, considering that understanding the nature of the muscle change, as well as the consequent functional effect, might orient the most appropriate rehabilitation treatment. For instance, it has been shown that muscle metabolic dysfunction is related to the impairment of walking in MS, so oxidative capacity and muscle endurance should be identified as potential therapeutic targets for interventions aimed at improving gait function [32,33,34].

Recent studies on skeletal muscle modification in MS are summarized in Table 2.

## 5. Parkinson’s Disease-Induced Skeletal Muscle Modifications

Weakness, low muscle power and fatigue are common in PD and can dramatically affect motor function and quality of life. As a result, patients with PD suffer from a severe sedentary lifestyle and have a lower functional capacity which is likely to aggravate the deleterious effects of the primary disease. Alterations in the central or peripheral nervous system controlling skeletal muscles could result in changes in the muscle tissue of PD patients. From previous, to more recent studies, it appears that the muscle modifications in PD are very heterogeneous and occur in a muscle-specific manner, depending on the duration, severity and subtypes of the disease [35,36].

Kelly et al. revealed a higher distribution and larger type I CSA myofibers and greater type II myofiber size heterogeneity on vastus lateralis biopsies. Authors speculated that the large type I fibers in PD might have resulted from a compensatory mechanism, an attempt to restore or retain the muscle mass, in response to preferential type II MU loss [37]. The results agreed with previous findings, suggesting that changes in muscle fibers might result from hypomobilization, causing disused myofibers atrophy [38,39], with a compensatory hypertrophy in the remaining fibers.

On the other hand, the apparent type I hypertrophy might result from higher levels of type I MU activity in PD, in which there is a selective use of low-threshold tonic MU, while high-threshold MU remain more inactive [36,40,41].

Mu et al. confirmed such muscular modifications also in pharyngeal muscle of PD patients: their sample showed altered fiber morphology and enzyme-histochemical activity, as well as fiber types grouping, reduced diameters, atrophy and denervation. The PD pharyngeal muscles showed a slow phenotype compared to HC, since the muscles analyzed had a decrease in both type I/IIA and IIA fibers, with an increase in the percentage of type I fibers compared to HC. Authors speculated that neurogenic myofiber atrophy, due to a PD-related loss of functioning motoneurons, and degenerative alterations in the peripheral nerves innervating pharyngeal muscles might play a pathogenic role in development of muscular disfunction in PD [42].

Wrede et al. suggest that myopathological changes found in paravertebral muscle of camptocormic PD patients might be related to a proprioceptive dysregulation [43]. Their described myopathic pattern was similar to that observed after experimental tenotomy [44]. Authors stated that experimental lesions depended on disturbances of muscle tension reflex mechanisms, but the reflexes needed to be functionally intact. Based on this finding, they speculated that muscular changes leading to camptocormia may also be related to a proprioceptive dysregulation [43].

The muscle oxidative metabolism appears to be impaired too, as demonstrated by a dual cohort study conducted by Saiki et al. [45]. The analysis pointed out a significant reduction in serum long-chain acylcarnitines (LCACs), at least at the onset of PD. Authors hypothesized that it might result from primary changes in skeletal muscle, not related to exercise and medication. Since serum LCACs originate mainly from the early stages of β-oxidation in skeletal muscle, the authors did not exclude that there might already be a mild skeletal muscle atrophy in the early stages of the disease [45]. Previous studies have already suggested that mitochondrial dysfunctions are associated with the pathogenesis of PD and mitochondrial respiratory dysfunction has been reported in skeletal muscle biopsy samples of PD patients [46,47]. Saiki et al. suggest that muscle modification occurs before the onset of PD typical signs and symptoms, so it might allow for early diagnosis in a pre-symptomatic stage [45]. Additionally, Di Martino et al. highlighted an impaired muscle oxidative efficiency in PD subjects, demonstrated by increased hematic lactate values during and after a submaximal incremental exercise on treadmill. This finding allowed the programming of an intensive rehabilitation program on a treadmill that showed a beneficial effect on muscle oxidative metabolism, endurance and balance, confirming the focal role of rehabilitation in patients with PD [48].

From the earliest clinical stage of PD, muscle atrophy can be observed and further emerges when PD-associated neuropathology in the muscle has advanced sufficiently to increase weakness and frailty [49]. Accordingly, muscle loss and increased fat content in PD patients are likely due to their reduced motor function. Furthermore, their analysis demonstrated that changes in higher intramuscular fat content in PD patients were not accompanied by noticeable changes in the CSA in analyzed regions; the same result was presented in a previous study of sarcopenia due to aging, suggesting a common pathogenesis [50].

In PD, it is general highlighted an impairment in type I fiber that, against a structural hypertrophy likely compensative to type II fiber loss, does not exhibit an adequate functional capacity. On this basis, rehabilitative treatment represents a valid tool in promoting skeletal muscle adaptations in PD, even if no solid studies on muscle metabolic features are still available.

Studies analyzing skeletal muscle status in PD over the past 10 years are summarized in Table 3.

## 6. Conclusions

In conclusion, in this review, we have tried for the first time, to the best of our knowledge, to summarize the current findings on muscle changes induced by the most common neurological diseases. These data underline the central role of skeletal muscle as a target of training interventions, especially in a long-term management. Although great strides have been made in studying possible changes in muscle composition after the onset of an acquired neurological disease, large gaps remain in this research area. To date, different technologies are useful for analyzing and monitoring changes that occur over time directly and indirectly (sEMG, PET, RMI, NIRS, DXA, Ultrasonography), but the most accurate indication of muscle composition remains the muscular biopsy, also with the limit of only a small sampling of muscle fiber. The research results are often conflicting due to the different methodologies and the heterogeneity of the samples involved. However, in MS e PD a specific involvement of type I fibers seems to be present, particularly for the metabolic properties in MS and structural aspects in PD, while for stroke a definitive conclusion cannot be assumed. Muscle tissue remains difficult to study as its adaptations may depend not only on the occurrence of damage but also on physiological differences in individual muscles or muscle groups and their function.

The lack of this knowledge points out the imperative need for future research, in which a specific patients’ stratification, rather than the use of homogeneous evaluation protocols, could allow the muscular modification in CNS disorders to be better defined. This know-how could best address the rehabilitative treatment of these pathologies in terms of timing, intensity and modality of the treatment approaches. Therefore, this could allow to a push towards a more customized rehabilitation in which patients are considered in a comprehensive manner in order to gain the optimal recovery.

## Figures and Tables

**Table 1:** Skeletal muscle modifications in Stroke (2010–2020).

Authors, Year	Type of Study	Type of Analysis	Analyzed Muscle	Analyzed Side	*n* Subjects (Male)	Mean Age in Years (±SD)	SP Characteristics	Mainly Findings
Kallenberg and Hermens 2011 [18]	Case-Control	HD-sEMG	Biceps Brachii	SP-Affected side HC-Dominant side	38 SP 18 (12) HC 20 (5)	SP 64 HC 59	Mean time since stroke: 2 years Affected side: 11 left, 7 right FMS: 35.5 Ashworth score: 2	RMS-MUAP was significantly larger in stroke than in HC. The Ashworth score was negatively correlated with RMS in active movement. The FMS was positively correlated to RMS-MUAP during passive movement.
Ryan, Ivey, Prior, et al. 2011 [8]	Cross-sectional	Muscle Biopsy	Vastus lateralis	Bilateral	15 SP 15 (66%)	SP 65 (±2)	Mean time since stroke: 8 years (±2)	Myostatin levels were 40% higher in the paretic than the non-paretic muscle.
Triandafilou and Kamper 2012 [9]	Case-Control	US	Muscles of the index finger	Bilateral	35 SP 25 (16) HC 10 (8)	SP NS (range 45–65 years) HC NS (range 45–65 years)	Mean time since stroke: 2,6 years (range 2–4) Affected side: 12 left, 13 right	Muscle size in the paretic hand was significantly reduced respect to non-paretic. There was no significant difference in size between SP non-paretic hand muscles and HC dominant hand. Size reduction in paretic limb was 12% beyond what would be expected in HC non-dominant limb. The extent of atrophy in a paretic limb is smaller for a previously dominant.
Oi, Itoh, Tobimatsu et al. 2015 [16]	Case-Control	PET	Gluteal Tight and lower leg muscles	SP-BilateralHC-Right	12 SP 8 (6) HC 11 (11)	SP 56 HC 26	Mean time since stroke: 2,6 years (range 1.3–4.2) Affected side: 3 left, 5 right Brunnstrom recovery stage at lower extremity: Level III: 6 patients Level VI: 2 patients	GUL of paretic lower leg muscles was significantly smaller than those of non-paretic limb and HC. In non-paretic limb of SP, GUL of lower leg muscles was larger than that of the thigh. GUL of medial hamstring and posterior tibial muscles were larger in SP non-paretic limb, respect to right limb of HC.
Hu, Suresh, Rymer et al. 2015 [20]	Cross-sectional	sEMG	First dorsal interosseous	Bilateral	14 SP 14 (6)	SP 60.5 (±5.9)	Mean time since stroke: 8.5 years (range 1–23) Affected side: 5 left, 9 right FMS: 39.14 (±21.05)	In paretic muscles there was a disruption of recruitment organization. The paretic muscles showed a weak relationship between recruitment order and MU size. The range of recruitment force thresholds in the paretic muscle was clustered to lower values and did not increase with voluntary muscle contraction, as compared with contralateral muscles.
Li, Fisher, Rymer, and 2016 [17]	Cross-sectional	sEMG	First dorsal interosseous	Bilateral	12 SP 12 (8)	SP 64 (±2)	Mean time since stroke: 10.2 years (±2.5)Affected side: 3 left, 9 right FMS: 44.08 (±21.03)	A significant reduction in MU number was estimated in paretic first dorsal interosseous muscles compared with the contralateral side. Paretic muscles have larger MUAPs.
Severinsen, Dalgas, Overgaard et al. 2016 [10]	Cross-sectional	Muscle biopsy	Rectus femoris	Bilateral	36 SP 36 (NS)	SP 68 (expressed as median)	Median time since stroke: 17 months (range 8–36) FMS: 74 (median, range 15–97)	On the paretic side compared with the non-paretic side it is evident a lower proportion of slow type I fibers and a higher proportion of fast fatigable type IIX fibers; there was a significantly smaller muscle fiber size and a reduced oxidative activity.
Klaer, Mahler, Scherbakov et al. 2016 [15]	Case-Control	Blood and micro-dialysis samples	Vastus lateralis (micro-dialysis)	Bilateral	17 SP 9 (9) HC 8 (8)	SP 62 (±8) HC 58 (±5)	Ischaemic/haemorrhagic aetiology: 8/1	Muscular metabolic properties did not differ between paretic and non-paretic leg. Glycolytic activity was higher in SP vs. HC, with increased lactate production.
Conrad, Qiu, Hoffmann et al. 2017 [21]	Case-Control	sEMG	Flexor digitorum superficialis; Extensor digitorum communis.	Bilateral	17 SP 12 (9) HC 5 (4)	SP 55 (±7) HC 28 (±7)	Mean time since stroke: 3.25 (±1.86) years Affected side: 5 left, 7 right Chedoke Stage: 3.41 (±1.24) - Stage 4 or 5 (moderate impairment) = 7pts - Stage 2 or 3 (severe impairment) = 5 pts	In both healthy subjects and stroke patients the MFCV was higher in the Extensor digitorum communis muscle as compared to the Flexor digitorum superficialis muscles. MFCV was lower in the paretic Extensor digitorum communis muscle than in the non-paretic one of SP.
Loureiro, Langhammer, Gjovaag et al. 2017 [12]	Case-Control	Ergospirometer	NA	NA	38 SP 28 (19) HC 10 (5)	SP 72.9 (±10.8) HC 69.6 (±10.9)	Ischaemic/haemorrhagic aetiology: 21/7 NIHSS: 3.5 (±2.7)	Carbohydrate oxidation is the main source of fuel during walking in PS. The higher consumption of carbohydrates can affect physical performance.
Ryan, Ivey, Serra et al. 2017 [7]	Case-Control	DXA	NA	NA	228 SP 190 (116) HC 38 (NS)	SP 63 (±1) HC 63 (±1)	NS	Sarcopenia in stroke varies between 14% and 18%. Paretic leg lean mass by DXA is lower than non-paretic leg lean mass in the total group.
Andersen, Jorgensen, Zeeman et al. 2017 [11]	Cross-sectional	Muscle biopsy	Vastus lateralis	Bilateral	8 SP 8 (6)	SP 46.6 (±4.4)	Mean time since stroke: 11 (±2) months Affected side: 5 left, 3 right	Paretic lower limbs had smaller muscle fiber size and lower type I and IIA and higher type IIX percentages than non-paretic lower limbs.

DXA: dual-energy X-ray absorptiometry; FMS: Fulg-Meyer Score; GUL: Glucose Uptake Level; HC: Healthy Control; HD-sEMG: High-Density surface Electromyography; MFCV: muscle fiber conduction velocity; MU: Motor Unit; MUAPs: Motor Unit Action Potentials; NA: Not applicable; NIHSS: National Institutes of Health Stroke Scale; NS: Not specified; PET: Positron Emission Tomography; pts: patients; RMS: root-mean-square; sEMG: surface Electromyography; SD: Standard Deviation; SP: Stroke Patients; US: Ultrasonography.

**Table 2:** Skeletal muscle modifications in Multiple Sclerosis (MS) (2010–2020).

Authors, Year	Type of Study	Type of Analysis	Analyzed Muscle	*n* Subjects (Male)	Mean Age in Years (±SD)	MS Characteristics	Mainly Findings
Scott, Hughes, Galloway, and Hunter 2011 [26]	Case-control	Surface EMG	Vastus lateralis	29 MS 15 (7) HC 14 (8)	MS 53.7 (±10.5) HC 54.6 (±9.6)	not available	MS patients have faster MFCV and reduced RMS during isometric contraction.
Malagoni, Felisatti, Lamberti et al. 2013 [28]	Case-control	NIRS	Gastrocnemius	50 MS 28 (16) HC 22 (13)	MS 42.7 (±14.0) HC 36.0 (±8.2)	MS Subtypes: RR 19 PP 9 Disease duration: 9.9 ± 6.3 years EDSS: 2.7 ± 1.6	rmVO2 is higher in MS and in low versus high performing patients.
Wens, Dalgas, Vandenabeele et al. 2014 [27]	Case-control	Muscle biopsy	Vastus lateralis	52 MS 34 (12) HC 18 (5)	MS 47.5 (±1.9) HC 45.7 (±1.7)	MS Subtypes: RR 26 CP 8 EDSS 2.5 ± 0.19	CSA of all fibers are smaller. Type II fibers experienced a larger atrophy, compared to type I. Type I proportion tended to be lower, type IIA proportion tended to be higher.
Hansen, Wens, Vandenabeele et al. 2015 [30]	Part1: Case-control Part2: Prospective observational	Muscle biopsy	Vastus lateralis	Part1: 24 MS 14 (10) HC 10 (6) Part2: 16 MS 9 (7) HC 7 (3)	Part1: MS 48 (±9) HC 48 (±8) Part2: MS 48 (±9) HC 47 (±10)	Part1: MS Subtypes: SP 3 RR 9 PP 2 EDSS 2.8 ± 1.2 Part2: SP 2 RR 6 PP 1 EDSS 2.6 ± 1.0	Basal muscle phospho-AMPKa and phospho-mTOR are increased in MS. There is a correlation between muscle phospho-AMPKa or phosphor-mTOR and whole-body fat mass, peak oxygen uptake, and EDSS.
Harp, McCully, Moldavskiy and Backus 2016 [29]	Case-Control	NIRS	Gastrocnemius	25 MS 16 (3) HC 9 (1)	MS 49.7 (±10.4) HC 40.1 (±9.8)	MS Subtypes: SP 4 RR 9 ND 3 Disease duration: 17 ± 11 years	MS had 40% lower mVO2max compared to the HC group. There are not significant correlations between walking speed andoxidative capacity

AMPKa: AMP-activated protein kinase; CP: chronic progressive; CSA: Cross sectional area; EDSS: Expanded Disability Status Scale; EMG: Electromyography; HC: Healthy Control; MFCV: Muscle fiber conduction velocity; MS: Multiple Sclerosis; mTOR: mammalian target of rapamycin; NA: Not applicable; ND: not determined; NIRS: Near-infrared spectroscopy; PP: Primary Progressive; RMS: Root Mean Square; rmVO2: resting muscle oxygen consumption; RR: Relapsing Remitting; SD: Standard Deviation; SP: secondary progressive.

**Table 3:** Skeletal muscle modifications in Parkinson’s Disease (PD) (2010–2020).

Authors, Year	Type of Study	Type of Analysis	Analyzed Muscle	*n* Subjects (Male)	Mean Age in Years (±SD)	PD Characteristics	Mainly Findings
Wredw, Margraf, Goebel et al. 2012 [43]	Case-Control	Muscle biopsy	Para-spinal	24 PD 14 (7) HC 10 (5)	PD 65.30 HC 66.5	Mean disease duration: 13.5 years Presence of Camptocormia	PD muscles show myopathic changes, type I fiber hypertrophy, loss of type II fibers, loss of oxidative activity, and acid phosphatase reactivity. Specific myopathic findings are: myofibrillar disorganization and Z-band streaming up to electron-dense patches/plaques.
Mu, Sobotka, Chen et al. 2012 [42]	Case-Control	Muscle biopsy	Pharyngeal Muscles	12 PD 8 (6) HC 4 (2)	PD 78.1 HC 77.5	Mean disease duration: 16.6 years H&Y stage (mean): 3.5 motorUPDRS: 41.0	Pharyngeal muscles of PD patients exhibited atrophic fibers, fiber type grouping, fast-to-slow myosin heavy chain transformation.
Kelly, Ford, Standaert et al. 2014 [37]	Case-Control	Muscle biopsy	Vastus lateralis	30 PD 15 (12) HC 15 (12)	PD 66.5 (±6) HC 65.3 (±6)	Mean disease duration: 4.4 years (range 1–16) H&Y stage: Stage 2 (10 pts) Stage 3 (5 pts)	PD muscles show higher distribution and larger CSA of type I myofibers and greater type II myofiber size heterogeneity.
Saiki, Hatano, Fujimaki et al. 2017 [45]	Double cohort	Blood samples	NA	1st cohort: 141 PD 109 (59) HC 32 (14) 2nd cohort: 190 PD 145 (70) HC 45 (23)	1st cohort: PD 67.3 (±9.99) HC 62.9 (±12.4) 2nd cohort: PD 67.5 (±10.2) HC 63.8 (±15.3)	1st cohort: Mean disease duration: 6.48 (±5.64) years motorUPDRS: 13.9 (±10.5) H&Y stage (mean): 2.15 (±0.91) 2nd cohort: Mean disease duration: 7.04 (±5.61) years motorUPDRS: 14.8 (±9.84) H&Y stage (mean): 2.09(±0.897)	PD patients show a decreased level of LCACs. Decreased levels of acylcarnitine, decreased ratio of acylcarnitine to fatty acid, and an increased index of carnitine palmitoyltransferase1 were identified in H&Y stage I of both cohorts, suggesting an initial β-oxidation suppression.
Di Martino, Tramonyi, Unti et al. 2018 [48]	Case-Control	Blood samples	NA	92 PD 60 (38) HC 32 (NS)	PD 67.4 (±8.8) HC 66.6 (±5.6)	Mean disease duration: 6.16 (±3.92) years H&Y stage (mean): 2.72 (±0.89) motorUPDRS: 31.05 (±13.71)	At rest, there are not significant difference in hematic lactate values between PD and HC. At the end of exercise and during the first recovery minutes, the lactate was significantly higher in PD than in HC. An intensive rehabilitation program reduced the lactate at the end of the exercise and during recovery.
Wang, Chen, Lin 2019 [49]	Case-Control	MRI	Bilateral Psoas and thigh muscles	45 PD 25 (5) HC 20 (4)	PD 63.6 (±5.54) HC 63.0 (±4.09)	Mean disease duration: 1.70 (±2.15) years motorUPDRS: 27.92 (±14.17) Modified H&Y (mean): 2.02 (±1.08)	PD patients show elevated degree of fatty replacement in the core muscles and bilateral thighs. Increased fatty content and decreased lean mass were highly associated with disease severity. Higher intramuscular fat content in PD patients are not accompanied by noticeable changes in the muscle CSA.

CSA: Cross-sectional area; HC: Healthy Control; H&Y: Hoehn and Yahr; LCACs: long-chain acylcarnitines; MRI: Magnetic resonance imaging; NA: Not applicable; PD: Parkinson’s Disease; SD: Standard Deviation; UPDRS: Unified Parkinson’s Disease Rating Scale.
